# Comparison of outcomes in patients with stage III versus limited stage IV non-small cell lung cancer

**DOI:** 10.1186/1748-717X-6-80

**Published:** 2011-06-30

**Authors:** Praveena Cheruvu, Su K Metcalfe, Justin Metcalfe, Yuhchyau Chen, Paul Okunieff, Michael T Milano

**Affiliations:** 1 Department of Radiation Oncology, University of Rochester Medical Center, Rochester, New York, USA; 2 Department of Radiation Oncology, University of Florida's College of Medicine, Gainesville, Florida, USA

**Keywords:** Stereotactic Body Radiotherapy, Oligometastases, Non-Small Cell Lung Cancer

## Abstract

**Background:**

Standard therapy for metastatic non small cell lung cancer (NSCLC) includes palliative systemic chemotherapy and/or radiotherapy. Recent studies of patients with limited metastases treated with curative-intent stereotactic body radiation therapy (SBRT) have shown encouraging survival. We hypothesized that patients treated with SBRT for limited metastases have comparable outcomes with those treated with curative-intent radiation for Stage III NSCLC.

**Methods:**

We retrospectively reviewed the records of NSCLC patients treated with curative-intent radiotherapy at the University of Rochester from 2000-2008. We identified 3 groups of patients with NSCLC: stage III, stage IV, and recurrent stage IV (initial stage I-II). All stage IV NSCLC patients treated with SBRT had ≤ 8 lesions.

**Results:**

Of 146 patients, 88% had KPS ≥ 80%, 30% had > 5% weight loss, and 95% were smokers. The 5-year OS from date of NSCLC diagnosis for stage III, initial stage IV and recurrent stage IV was 7%, 14%, and 27% respectively. The 5-year OS from date of metastatic diagnosis was significantly (p < 0.00001) superior among those with limited metastases (≤ 8 lesions) versus stage III patients who developed extensive metastases not amenable to SBRT (14% vs. 0%).

**Conclusion:**

Stage IV NSCLC is a heterogeneous patient population, with a selected cohort apparently faring better than Stage III patients. Though patients with limited metastases are favorably selected by virtue of more indolent disease and/or less bulky disease burden, perhaps staging these patients differently is appropriate for prognostic and treatment characterization. Aggressive local therapy may be indicated in these patients, though prospective clinical studies are needed.

## Background

Non-small cell lung cancer (NSCLC) is the leading cause of cancer mortality in the United States [1]. Patients with stage IV NSCLC typically have a poor prognosis, with a median survival of 8 months [2]. Palliative systemic therapy improves survival and disease control, though careful selection of patients suitable for systemic therapy is critical. Radiation treatment for symptomatic relief is a common approach utilized by many clinicians.

Stage IV NSCLC represents a heterogeneous stage grouping, with regard to the extent of disease spread, cancer burden (i.e. bulk), performance status, and other prognostic factors. As previously postulated by Drs. Hellman and Weichselbaum, the metastatic disease state lies on a spectrum rather than occupying a finite point [3]. Thus, the definition of metastatic disease could be refined in terms of limited versus extensive disease extent and/or disease bulk Patients with limited metastatic disease and good performance have been shown to have better outcomes following aggressive local treatments, i.e. resection, stereotactic body radiotherapy (SBRT), radiofrequency ablation [4].

In previous studies from the University of Rochester, we defined oligometastases as the presence of 5 or fewer clinically apparent metastases [5,6]. Recent studies, including our own, suggest that patients with limited metastases who receive curative-intent SBRT to metastatic sites, including patients with multiple organ involvement, have encouraging survival [7,8]. SBRT implies the use of hypofractionated, highly conformal external beam radiotherapy, utilizing technology to improve targeting accuracy, such as external or internal stereotactic fiducial markers and/or image guidance. Although data on SBRT treatment for limited metastases from breast cancer and colon cancer have been encouraging, there is limited outcome data following SBRT for metastatic NSCLC. We hypothesized that curative-intent SBRT for limited metastatic (i.e., select stage IV) NSCLC results in comparable outcomes relative to stage III NSCLC treated with aggressive curative intent treatment.

## Methods

We retrospectively reviewed the records of patients with NSCLC who were treated at the University of Rochester from 2000-2008 with curative-intent radiotherapy (with or without chemotherapy) for stage III NSCLC or SBRT for limited stage IV NSCLC, defined as metastatic disease treated with curative-intent. The study was approved by the University of Rochester Research Subjects Review Board.

Those patients with metastases limited in number and extent, clinically determined to be amenable to SBRT, were defined as having limited metastases. While previous prospective studies from our group [5,6] defined limited metastases/oligometastases as 5 or fewer lesions, in this study, six NSCLC patients with 6-8 metastatic lesions were considered to have limited metastatic NSCLC, as all radiographically apparent lesions were amenable to SBRT as defined by our previously published normal tissue tolerances for SBRT [9]. Several patients underwent repeat courses of SBRT for either additional lesions or for lesions that recurred after SBRT.

Patients with metastases from NSCLC to any organ(s), including brain, bone, liver, or lung were included in this study. Only patients with biopsy-proven NSCLC were included. Those with prior curative-intent treatment of metastatic tumors (i.e. surgery) were not excluded. Patients with new solitary lung lesions, questionable for metachronous stage I NSCLC versus solitary metastases were excluded.

The 7th AJCC Staging system was used to categorize patients into 3 groups: those who were stage III at initial diagnosis, those who presented with stage IV NSCLC, and those with stage I/II NSCLC who later developed recurrent stage IV disease. Among patients with Stage III NSCLC, metastatic recurrence was characterized as limited metastatic (defined above) or extensive metastatic (not amenable to curative-intent SBRT) disease.

All patients were assessed with diagnostic CT or PET/CT imaging to identify metastatic lesions. Brain metastases were assessed with MRI. In some patients bone metastases were identified with bone scan and/or MRI. Patients who had brain metastases were treated curatively with surgery and/or stereotactic radiosurgery. Those that received surgery for brain metastases had SBRT to other sites of metastatic disease. A variety of chemotherapeutic or radio-sensitizing agents were utilized.

The net gross tumor volume (GTV) was documented as the sum of GTVs of the primary and/or metastatic sites. The GTVs were based on the contoured volumes on the planning CT scan(s). Previously resected metastases were not included in the net tumor volume.

This study was approved by the University of Rochester Research Subjects Review Board.

### Conventional Radiotherapy

Stage III patients underwent a CT simulation for treatment planning purposes. Definitive radiotherapy was given concurrently with chemotherapy or as adjuvant treatment following surgery. A 3D conformal technique was utilized to shape the dose around the tumor volume with an appropriate volumetric expansion. The average dose prescribed was 60 Gy but ranged from 45 to 66 Gy.

#### SBRT Technique

SBRT was delivered through the Novalis Exac Trac^® ^System. All patients were immobilized during simulation and treatment using a vacuum cushion device. An end-expiratory or shallow breath-hold technique was used in conjunction with the ExacTrac^® ^patient positioning platform (BrainLAB^®^, AG, Heimstetten, Germany) to accurately reproduce patient set-up during treatments. The ExacTrac^® ^system consists of external body fiducial markers monitored in real time by two ceiling mounted infrared cameras. Treatment planning was performed with BrainLAB planning software. SBRT was delivered using conformal arcs, and the dose was prescribed to the isocenter with the 80% isodose line covering the planning target volume (PTV). The treatment volume included a 7-10 mm volumetric expansion of the GTV. Typically, the dose schedule was 50-60 Gy in 5-10 fractions. Depending on the location of the lesion and the dose volume histogram of the organs at risk, a more hypofractionated schedule was utilized in 5 fractions. Our institution has adapted a 10 fraction scheme of treating patients with SBRT to reduce potential toxicity while preserving control. Typically peripheral lung lesions were treated with 5 fractions to 50 Gy, while more central lung lesions, adrenal, liver, abdominal/pelvic lymph node metastases were treated with 10 fractions to 50 Gy.

#### Endpoints

The primary endpoint was overall survival (OS). OS was calculated in two manners: from the date of initial NSCLC diagnosis, as well as from the date of first metastases, to date of last follow-up or death, using Kaplan-Meier actuarial survival analyses. OS was measured from date of initial diagnosis to compare outcomes of limited stage IV and Stage III NSCLC patients. As an alternate measure of OS, the date of first metastases was used as a reference point to compare the outcomes of recurrent metastatic patients versus those who presented with initial limited metastatic disease. Univariate analyses (UVA) were performed with log-rank tests (for discrete variables) or Cox regression analyses (for continuous variables). Significant (p ≤ 0.05) variables on UVA were tested with multivariate analyses (MVA), using a Cox proportional hazards regression model. Chi square tests and ANOVA tests were used to compare patient and tumor characteristics between the subgroups. Stata version 9.2 was used for all data analyses. The retrospective nature of this study did not allow for detailed assessment of treatment toxicity; previous reports from our group have suggested minimal adverse effects after SBRT [10,11].

## Results

### Patient and disease characteristics

Patient and disease characteristics are summarized in Additional file 1. From 2000- 2008, 146 patients were eligible for analysis. Patient age ranged from 35 - 85 years (median 65 years). Eighty-eight percent had Karnofsky performance status (KPS) ≥ 80%, and 30% had > 5% weight loss at time of diagnosis. The majority of patients (95%) had a prior smoking history. One-hundred patients received chemotherapy during the course of their cancer treatment, and 22 patients underwent prior resection of their primary lung mass. Thirteen patients had curative resection of a metastatic lesion prior to their course of SBRT. Metastatic sites apparent at the time of SBRT for limited metastases included lung or thoracic lymph nodes (n = 22), liver (n = 13), adrenal glands (n = 16), bone (n = 7), retroperitoneal lymph node (n = 2), and CNS (n = 10). Thirteen patients developed brain metastases at the time of extensive metastatic progression (n = 13). The net GTV ranged from 0.9 - 877.1 ml (median 85.7 ml). Compared to stage III patients, those with initial limited Stage IV disease had less bulkier disease (median 93.9 ml vs. 76.5 ml respectively), though not significant (p = 0.27; Additional File 1).

There were a total of 94 patients (64.3%) categorized as stage III NSCLC, 38 patients (26.0%) with initial limited stage IV NSCLC and 14 patients (9.6%) with stage I/II NSCLC who progressed to limited metastatic stage IV. Fifty-five of the 94 patients with initial stage III NSCLC (74%) developed radiographically apparent metastases, of whom 44 developed limited metastatic stage IV disease (all with ≤5 lesions), treated with SBRT. The average time to recurrence for these Stage III patients was 14.2 months (range 3.2-45.2 months). Among those with Stage III and Stage IV disease all had a staging CT or MRI of head. All 38 patients with stage IV NSCLC had PET/CT while 90 of 93 patients with stage III NSCLC had PET/CT.

### Outcomes

**Figure 1:**
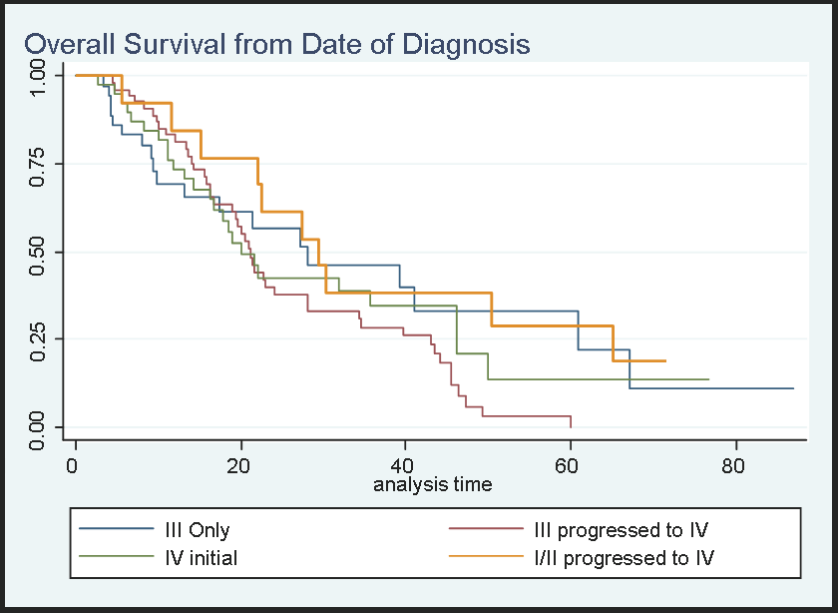
**represents the overall survival from date of diagnosis for the various stage groupings**. The blue line represents Stage III (whom did not progress to Stage IV, maroon line represents Stage III which progressed to Stage IV, green line represents the initial stage IV patients and lastly the gold line represents the Stage I/II patients that progressed to Stage IV.

The OS calculated from time of initial NSCLC diagnosis is presented in Additional file 2 and Figure 1. The 2-year and 5-year OS for all patients with stage III NSCLC was 43% and 7% respectively versus 43% and 14% for initial limited stage IV (p = 0.30).

**Figure 2:**
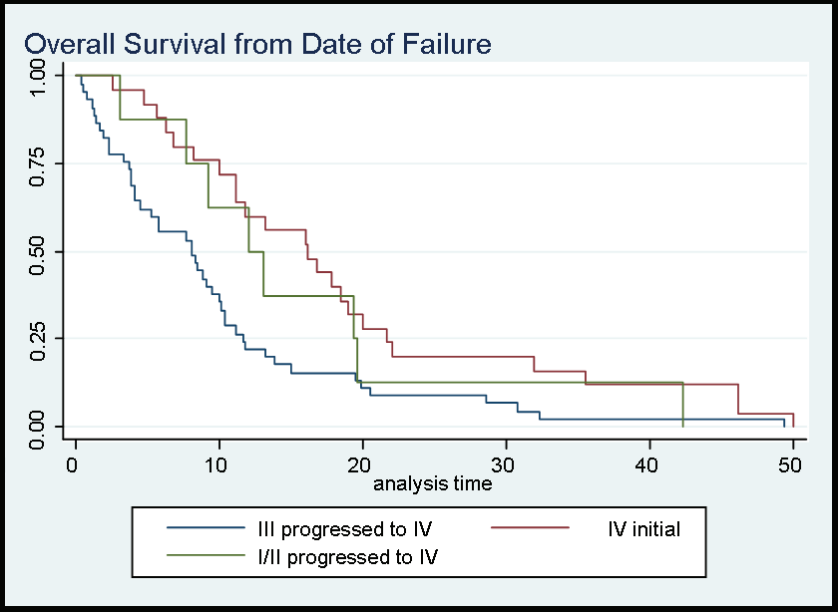
**represents the overall survival from date of failure**. The blue line represents Stage III which progressed to Stage IV, green line represents the Stage I/II patients that progressed to Stage IV, and lastly the maroon line represents initial stage IV patients.

The OS calculated from the date of diagnosis of metastatic disease is presented in Additional file 3 and Figure 2. Worse survival was observed in patients with initial stage I/II or Stage III NSCLC who developed metastases (5-year OS 0%, with no survivors beyond 4.1 years) versus those who initially presented with limited metastatic disease (5-year OS 14%, p = 0.003).

Among those with recurrent stage IV NSCLC (stage I/II, or III who progressed to stage IV) the patients who developed limited metastases had a 2-year and 5-year OS from date of metastatic progression of 25% and 0% respectively. In contrast, there were no survivors beyond 11.0 months among the 14 patients with stage I/II or III NSCLC who developed extensive metastatic disease (p < 0.00001). For patients with initial diagnosis of limited stage IV NSCLC, there was no significant difference among patients with ≤ 5 metastases versus 6-8 (p = 0.94).

Age, KPS, weight loss, smoking history, stage and net GTV were assessed for affect on OS from date of initial NSCLC diagnosis as shown in Additional file 4. On univariate analysis of OS from date of initial NSCLC diagnosis, GTV at time of radiation for initial stage III or IV NSCLC (p < 0.002) was the only variable which was statistically. Stage III versus limited metastatic Stage IV trended (p = 0.078) towards worse OS. MVA models for OS were also assessed with the same variables; net GTV remained a statistically significant (p = 0.001) factor for predicting OS. MVA using only stage and GTV did not change the results. MVA models for OS from date of metastases treated with SBRT were assessed, with the same variables. Net GTV of metastases treated with SBRT was not statistically significant (p = 0.39).

## Discussion

The standard treatment for metastatic NSCLC is systemic therapy. However, in those patients with metastases limited in number and location, local surgical or ablative treatments have shown to be beneficial. Series describing surgical metastectomy have the largest patient numbers and longest follow up [12-14]. Surgical metastatectomy is proposed to be a curative therapy, resulting in prolonged disease-free survival [15]. Recently, there is an expanding experience with stereotactic radiotherapy as effective local therapy for metastatic lesions from any primary cancer. Local control rates of 60-90% have been reported for metastatic tumors involving spine, lung, and liver [16-20].

In this study, patients with initial stage IV NSCLC treated with aggressive local therapy had a higher 5-year OS when compared to stage III NSCLC patients treated with curative intent (14% and 7%, respectively). Although our 5-year survival rate of 7% for Stage III NSCLC patients is lower than what is reported in Phase II-III studies of definitive radiation, our Stage III patients represent an unselected cohort of all patients seen in our clinic, many of whom had adverse prognostic factors such as poor performance status and/or significant weights loss, unlike most patients receiving chemoradiation/radiation on prospective studies [21,22]. Most of our patients with Stage III NSCLC had bulky disease, and many would not qualify for prospective studies because of poor performance status and/or > 5% weight loss (Additional file 1). The 5-year OS rate of patients presenting with limited stage IV NSCLC, which is a selected cohort of patients with disease amenable to SBRT, was similar to those with stage III NSCLC that did not metastasize, suggesting that a number of patients in AJCC stage IV group would be more appropriately classified as stage III NSCLC in future AJCC staging. These data support the hypothesis that select patients with stage IV NSCLC have excellent outcomes comparable to unselected patients with stage III disease when treated aggressively with SBRT.

Patients with initial limited stage IV NSCLC fared better than those with stage I/II or III NSCLC who later progressed to stage IV (5-year OS 22% versus 0% from time of metastatic diagnosis). Perhaps limited metastases presenting as Stage IV NSCLC is biologically more responsive to cancer therapy than recurrent disease.

There may be a period during which local therapy may be most beneficial for patients who initially present with limited metastatic disease. Studies have shown that effective chemotherapy can reduce the number and sites of metastatic tumors at which time, local therapy may be considered [23-25]. Rusthoven et al reported that after NSCLC patients receive systemic chemotherapy for limited metastases, the majority (64%) progressed with local failure only; the time to progression in these patients was 3 - 4 months, thereby providing a critical window during which curative local therapy could be offered. Researchers from the University of Chicago analyzed the patterns of recurrence among 38 patients with stage IIIB and IV NSCLC who were enrolled on a Phase II trial of oxaliplatin and paclitaxel [24]. Half of the patients (19/38) had stable disease or progressed in the original sites only without developing new lesions after the completion of chemotherapy. Seventeen patients who had limited disease (≤ 4 metastatic sites) at initial presentation had a higher propensity to have stable disease or progress only at the initially involved sites. These studies demonstrate that there is a point at which progression of metastatic disease (i.e., development of new lesions) may be suppressed by systemic chemotherapy, allowing for aggressive local treatment to address residual disease.

The role of combined modality treatment for metastatic NSCLC patients was investigated by Khan, et al in a retrospective series of 23 patients with oligometastatic (1-2 sites) NSCLC disease [25]. These patients received curative treatment for their primary thoracic disease and subsequently received local treatment for their metastatic sites. At 28 months, overall survival was 22% with median survival of 20 months. In our study, patients with initial limited metastatic NSCLC treated with SBRT had a 2-year OS of 48% - this difference could be attributed to differences in patients or cancer characteristics or perhaps recent improvements in systemic chemotherapy and local treatment techniques. With a combined modality approach, the potential for further metastatic disease dissemination may be minimized, thus potentially translating to improved OS.

Among patients with recurrent metastatic NSCLC, those with limited metastases (2-year survival of 24%) had superior outcomes compared to those with extensive disease (no survivors beyond 11.0 months). This is consistent with the hypothesis that a larger tumor burden may predict for increased risk of local failure and metastatic potential. Among patients with limited metastases treated with curative intent SBRT, there were not significant differences in survival between those with ≤ 5 metastases versus 6-8 metastases, and in fact their survival is equivalent at 47 months. While small patient numbers limit analyses, this suggests that other variables such as tumor bulk and/or tumor location are more critical than number of lesions. Previous studies from our group of patients who underwent SBRT for limited metastases (not necessarily from NSCLC) [10,11,26] have shown net GTV to significantly impact disease control and survival outcomes.

In patients with non-metastatic NSCLC, numerous studies suggest that tumor burden correlates with prognosis [27-30]. In a small cohort of 19 patients with stage I-III NSCLC, Lee et al reported that tumor volume > 25 cc measured on PET CT scan was associated with increased risk of disease progression and was postulated to be a poor prognostic factor in lung cancer. Similarly, Bradley et al. demonstrated the prognostic value of GTV determined on planning CT scans to be associated with OS and local tumor control. This correlation appears to persist for patients with metastatic NSCLC [31] as reported by Oh et al and our present observations in which net GTV predicted for improved OS (MVA p < 0.0001). However, GTV of metastatic sites treated with SBRT was not significantly correlated with OS. The GTV of metastatic sites included patients with initial Stage III who developed limited metastases.

Weaknesses of our study include the retrospective nature of the analyses, heterogeneity of patient treatment, including various systemic therapy regimens (which was not presented), heterogeneity of involved metastatic sites (which was not analyzed) and, for some patient subgroups, relatively small patient numbers. Strengths of our study include an overall relatively large study population (particularly unselected Stage III patients and select patients with limited metastases from NSCLC) allowing for the analysis of several prognostic variables.

Stage IV NSCLC represents a heterogeneous patient population. Those patients with limited tumor burden in terms of volume and number of lesions are amenable to, and may benefit from focal ablative therapy to known sites of disease. In our study, select patients with limited stage IV NSCLC treated with curative-intent SBRT, have similar outcomes compared to those unselected patients with stage III NSCLC. While the benefit of SBRT (or other ablative therapies) for limited metastatic NSCLC has not been definitively proven from controlled randomized studies, even with the possibility of no benefit from ablative therapies, select patients with Stage IV NSCLC fare relatively well, and perhaps warrant unique consideration in future AJCC stage groupings. It reasonable to postulate that these patients fare well by virtue of a less indolent disease process in conjunction with a therapeutic benefit from ablative therapy. Given the relatively poor prolonged NSCLC disease control from systemic therapy, it is difficult to postulate that ablative therapies are not benefiting patients. Nonetheless, the favorable outcomes reported here warrant consideration of further investigation of local treatment for patients with limited tumor burden. The University of Chicago has launched a randomized Phase II trial of patients with 1-5 metastases from NSCLC to assess the survival with the addition of hypofractionated image guided radiotherapy concurrently with docetaxel and cisplatin. The NCCTG is also randomizing patients with 1-5 metastases from NSCLC, to undergo or not undergo radiation (with standard fractionation) to metastatic sites.

## Conflicts of Interest Notification

The authors declare that they have no competing interests.

## Authors' contributions

PC and SM participated in creation of database and input of data. PC drafted manuscript. JM and PEC performed the statistical analysis. YC, PO and MT treated the patients on study. MT participated in design of study and coordination and helped to draft the manuscript. All authors read and approved the final manuscript.

## Supplementary Material

Additional file 1 **Table S1- patient characteristics**. Click here for file

Additional file 2 **Table S2 - overall survival from date of diagnosis**. Click here for file

Additional file 3 **Table S3 - overall survival from date of metastases**. Click here for file

Additional file 4 **Table S4 - univariate & multivariate analysis for os from date of initial nsclc diagnosis**. Click here for file

## References

[B1] Jemal A Siegel R Xu J Ward E Cancer Statistics, 2010 CA Cancer J Clin 2010 60 5 277 300 Epub 2010 Jul 7 10.3322/caac.20073 20610543

[B2] Greene FL Page DL Fleming ID Fritz A Balch CM Haller DG Morrow M AJCC Cancer Staging Manual 2002 6 New York: Springer

[B3] Hellman S Weichselbaum RR Oligometastases J Clin Oncol 2005 13 8 10 10.1200/JCO.1995.13.1.8 7799047

[B4] Timmerman RD Bizekis CS Pass HI Fong Y Dupuy DE Dawson LA Lu D Local Surgical, Ablative and Radiation Treatment of Metastases CA Cancer J Clin 2009 59 145 170 10.3322/caac.20013 19364702

[B5] Milano MT Katz AW Muhs AG Philip A Buchholz DJ Schell MC Okunieff P A Prospective Pilot study of Curative Intent Stereotactic Body Radiotherapy in Patients with 5 or fewer Oligometastatic Lesions Cancer 2008 112 650 658 10.1002/cncr.23209 18072260

[B6] Milano MT Katz AW Schell MC Philip A Okunieff P Descriptive Analysis of Oligometastatic Lesions Treated with Curative-Intent Stereotactic Body Radiotherapy Int J Radiat Oncol Biol Phys 2008 72 5 1516 22 10.1016/j.ijrobp.2008.03.044 18495378

[B7] Kao J Packer S Vu HL Schwartz ME Sung MW Stock RG Lo YC Huang D Chen SH Cesaretti JA Phase 1 Study of Concurrent Sunitinib and Image Guided Radiotherapy followed by Maintenance Sunitinib for Patients with Oligometastases: Acute Toxicity and Preliminary Response Cancer 2009 115 15 3571 80 10.1002/cncr.24412 19536893 PMC4370266

[B8] Salama JK Chmura SJ Mehta N Yenice KM Stadler WM Vokes EE Haraf DJ Hellman S Weichselbaum RR An Initial Report of Radiation Dose-Escalation Trial in Patients with One to Five Sites of Metastatic Disease Clin Cancer Res 2008 14 160 5255 9 18698045 10.1158/1078-0432.CCR-08-0358

[B9] Milano MT Constine LS Okunieff P Normal Tissue Toxicity after Small Field Hypofractionated Stereotactic Body Radiation Radiat Oncol 2008 3 36 10.1186/1748-717X-3-36 18976463 PMC2596155

[B10] Katz AW Carey-Sampson M Muhs AG Milano MT Schell MC Okunieff P Hypofractionated Stereotactic Body Radiation Therapy (SBRT) for Limited Hepatic Metastases Int J Radiat Oncol Biol Phys 2006 67 793 798 17197128 10.1016/j.ijrobp.2006.10.025

[B11] Okunieff P Petersen AL Philip A Milano MR Katz AW Boros L Schell MC Stereotactic Body radiation Therapy (SBRT) for Lung Metastases Acta Oncol 2006 45 808 817 10.1080/02841860600908954 16982544

[B12] Choti MA Sitzmann JV Tiburi MF Sumetchotimetha W Rangsin R Schulick RD Lillemoe KD Yeo CJ Cameron JL Trends in Long-Term Survival Following liver resection for Hepatic Colorectal Metastases Ann surg 2002 235 759 766 10.1097/00000658-200206000-00002 12035031 PMC1422504

[B13] Sternberg DI Sonett jr Surgical Therapy of Lung Metastases Semin Oncol 2007 133 967 972 10.1053/j.seminoncol.2007.03.004 17560980

[B14] Tanvetyanon T Robinson L Schell MJ Strong VE Kapoor R Coit DG Bepler G Outcomes of Adrenalectomy for Isolated versus Metachronous Adrenal Metastases in Non-Small- Cell Lung Cancer: A Systematic Review and Pooled Analysis J Clin Oncol 2008 26 1142 1147 10.1200/JCO.2007.14.2091 18309950

[B15] Pfannschmidt J Dienemann H Surgical Treatment of Oligometastatic Non-Small Cell Lung Cancer Lung Cancer 2010 69 251 258 10.1016/j.lungcan.2010.05.003 20537426

[B16] Rusthoven KE Kavanagh BD Burri SH Chen C Cardenes H Chidel MA Pugh TJ Kane M Gaspar LE Schefter TE Multi-institutional phase I/II trial of stereotactic body radiation therapy for lung metastases J Clin Oncol 2009 27 1579 1584 10.1200/JCO.2008.19.6386 19255320

[B17] Kavanagh BD McGarry RC Timmerman RD Extracranial Radiosurgery (Stereotactic Body Radiation Therapy) for Oligometastases Semin Radiat Oncol 2006 16 2 77 84 10.1016/j.semradonc.2005.12.003 16564443

[B18] Lo SS Sahgal A Wang JZ Mayr NA Sloan A Mendel E Chang EL Stereotactic Body Radiation Therapy for Spinal Metastases Discov Med 2010 9 47 289 96 20423672

[B19] Rusthoven KE Kavanaugh BD Cardenes H Stieber VW Burri SH Feigenberg SJ Chidel MA Pugh TJ Franklin W Kane M Gaspar LE Schefter TE Multi-institutional Phase I/II Trial of Stereotactic Body Radiation Therapy for Liver Metastases J Clin Oncol 2009 27 10 1572 8 10.1200/JCO.2008.19.6329 19255321

[B20] Chang EL Shiu AS Mendel E Mathews LA Mahajan A Allen PK Weinberg JS Brown BW Wang XS Woo SY Cleeland C Maor MH Rhines LD Phase I/II study of stereotactic body radiotherapy for spinal metastasis and its pattern of failure J Neurosurg Spine 2007 7 151 60 10.3171/SPI-07/08/151 17688054

[B21] Curran WJ Scott CB Langer CJ Komaki R Lee S Hauser S Movsas B Wasserman T Sause W Cox JD Long-term benefit is observed in a phase III comparison of sequential vs concurrent chemo-radiation for patients with unresected stage III NSCLC: RTOG 9410 Proc Am Soc Clin Oncol(ASCO) 2003 abstr. #2499 Chicago 621

[B22] Gandara DR Chansky K Albain KS Leigh BR Gaspar LE Lara PN Burris H Gumerlock P Kuebler JP Bearden JD Crowley J Livingston R Consolidation Docetaxel After Concurrent Chemoradiotherapy in Stage IIIB Non-Small-Cell Lung Cancer: Phase II Southwest Oncology Group Study S9504 JCO 2003 21 10 2004 2010 10.1200/JCO.2003.04.197 12743155

[B23] Rusthoven KE Hammerman SF Kavanagh BD Birtwhistle MJ Stares M Camidge DR Is there a Role for Consolidative Stereotactic Body Radiation Therapy Following First-Line Systemic Therapy for Metastatic Lung Cancer? A Patterns-of-Failure Analysis Acta Oncologica 2009 48 578 583 10.1080/02841860802662722 19373699

[B24] Mehta N Mauer AM Hellman S Haraf DJ Cohen EE Vokes EE Weichselbaum RR Analysis of Further Disease Progression in Metastatic NSCLC: Implications for Locoregional Treatment Int Journal of Oncology 2004 25 1677 1683 15547705

[B25] Khan AJ Mehta PS Zusag TW Bonomi PD Penfield Faber L Shott S Abrams RA Long term Disease Free Survival Resulting from Combined Modality Management of Patients Presenting with Oligometastatic NSCLC Radiotherapy and Oncology 2006 81 163 167 10.1016/j.radonc.2006.09.006 17050016

[B26] Chawla S Chen Y Katz AW Muhs AG Philip A Okunieff P Milano MT Stereotactic Body Radiotherapy for Treatment of Adrenal Metastases Int J Radiat Oncol Biol Phys 2009 75 1 71 5 10.1016/j.ijrobp.2008.10.079 19250766

[B27] Lee P Weerasuriya DK Lavori PW Hara W Maxim PD Le QT Wakelee HA Donington JS Graves EE Loo BW Metabolic Tumor Burden Predicts for Disease Progression and Death in Lung Cancer IJROBP 2007 69 2 328 333 10.1016/j.ijrobp.2007.04.036 17869659

[B28] Bradley J Ieumwananonthachai N Purdy JA Wasserman TH Lockett MA Gramham MV Perez CA Gross Tumor Volume Critical Prognostic Factor in Patients Treated with Three Dimensional Conformal Radiotherapy for NSCLC IJROBP 2002 52 1 49 57 10.1016/s0360-3016(01)01772-2 11777621

[B29] Basaki K Abe Y Aoki M Kondo H Hatayama Y Nakaji S Prognostic factors for survival in Stage III non-small-cell-lung cancer treated with definitive radiation therapy: impact of tumor volume IJROBP 2006 64 449 454 10.1016/j.ijrobp.2005.07.967 16226400

[B30] Nishio W Sakamoto T Uchino K Yuki T Nakagawa A Tsubota N Effect of Tumor Size on Prognosis in Patients with Non Small Cell Lung Cancer: the Role of Segmentectomy as a Type of Lesser Resection J Thoracic Cardiovasc Surg 2005 129 87 93 10.1016/j.jtcvs.2004.04.030 15632829

[B31] Oh Y Taylor S Bekele BN Debnam JM Allen PK Suki D Sawaya R Komaki R Stewart DJ Karp DD Number of Metastatic Sites is a Strong Predictor of Survival in Patients with Nonsmall Cell Lung Cancer With or Without Brain Metastases Cancer 2009 115 2930 2938 10.1002/cncr.24333 19441110

